# Machine learning and deep learning to predict mortality in patients with spontaneous coronary artery dissection

**DOI:** 10.1038/s41598-021-88172-0

**Published:** 2021-04-26

**Authors:** Chayakrit Krittanawong, Hafeez Ul Hassan Virk, Anirudh Kumar, Mehmet Aydar, Zhen Wang, Matthew P. Stewart, Jonathan L. Halperin

**Affiliations:** 1 Section of Cardiology, Baylor College of Medicine, 1 Baylor Plaza, Houston, TX 77030 USA; 2 Icahn School of Medicine at Mount Sinai, The the Zena and Michael A. Wiener Cardiovascular Institute, Mount Sinai Heart, New York, NY USA; 3 Department of Cardiovascular Diseases, Case Western Reserve University, University Hospitals Cleveland Medical Center, Cleveland, OH USA; 4 Heart and Vascular Institute, Cleveland Clinic, Cleveland, OH USA; 5 Department of Computer Science, Kent State University, Kent, OH USA; 6 Robert D. and Patricia E. Kern Center for the Science of Health Care Delivery, Mayo Clinic, Rochester, MN USA; 7 Division of Health Care Policy and Research, Department of Health Sciences Research, Mayo Clinic, Rochester, MN USA; 8 The Institute of Applied and Computational Sciences, Harvard University, Boston, MA USA; 9 School of Engineering and Applied Sciences, Harvard University, Boston, MA USA

**Keywords:** Interventional cardiology, Machine learning, Statistical methods

## Abstract

Machine learning (ML) and deep learning (DL) can successfully predict high prevalence events in very large databases (big data), but the value of this methodology for risk prediction in smaller cohorts with uncommon diseases and infrequent events is uncertain. The clinical course of spontaneous coronary artery dissection (SCAD) is variable, and no reliable methods are available to predict mortality. Based on the hypothesis that machine learning (ML) and deep learning (DL) techniques could enhance the identification of patients at risk, we applied a deep neural network to information available in electronic health records (EHR) to predict in-hospital mortality in patients with SCAD. We extracted patient data from the EHR of an extensive urban health system and applied several ML and DL models using candidate clinical variables potentially associated with mortality. We partitioned the data into training and evaluation sets with cross-validation. We estimated model performance based on the area under the receiver-operator characteristics curve (AUC) and balanced accuracy. As sensitivity analyses, we examined results limited to cases with complete clinical information available. We identified 375 SCAD patients of which mortality during the index hospitalization was 11.5%. The best-performing DL algorithm identified in-hospital mortality with AUC 0.98 (95% CI 0.97–0.99), compared to other ML models (*P* < 0.0001). For prediction of mortality using ML models in patients with SCAD, the AUC ranged from 0.50 with the random forest method (95% CI 0.41–0.58) to 0.95 with the AdaBoost model (95% CI 0.93–0.96), with intermediate performance using logistic regression, decision tree, support vector machine, K-nearest neighbors, and extreme gradient boosting methods. A deep neural network model was associated with higher predictive accuracy and discriminative power than logistic regression or ML models for identification of patients with ACS due to SCAD prone to early mortality.

## Introduction

Machine learning (ML), a branch of artificial intelligence (AI), is applicable to risk modeling in medicine. Deep learning (DL) is a form of ML typically implemented through multi-layered neural networks to interpret and classify complex datasets and enhance clinical decision-making. When applied to big data in medicine, ML and DL, particularly convolutional neural networks (ResNet, GoogLeNet, or VCG families) are well suited to clinical image recognition and estimation of prognosis in large datasets^1, 2^. Information about the performance of this technology in smaller datasets, such as rare diseases or patient cohorts with heterogeneous conditions, infrequent events, or comorbidities that cause competing risks is considerably more limited. ML may perform poorly at predicting low frequency events, such as those with an incidence < 10%, and scant data are available on the performance of DL in heterogeneous populations with a low frequency of events^3^. Spontaneous coronary artery dissection (SCAD) is a heterogeneous condition (high noise) and uncommon cause of acute coronary syndrome (ACS) associated with low mortality (infrequent events)^4, 5^. Although there have been other ML-based approaches for predicting mortality risk in patients with ACS, predictors of early mortality in patients with SCAD have not been identified. We hypothesized that ML and particularly DL models could predict in-hospital mortality in patients with ACS due to SCAD, based on information extracted from electronic health records (EHR), with greater accuracy that conventional risk classification methods^6, 7^. Accordingly, we compared the performance of conventional logistic regression, ML modeling, and custom-built DL models to predict mortality in patients with SCAD using data from the EHR of a large urban health system.

## Methods

### Study population

We identified patients with a principal diagnosis of SCAD by querying the entire Mount Sinai Health System EHR for the period from January 1, 2008, to December 31, 2018, using International Classification of Diseases (ICD) 9 (414.12) and 10 codes (I25.42) for SCAD diagnosis, including only those with procedural (CPT) codes for coronary angiography and/or percutaneous coronary intervention, and excluding those with diagnoses indicating iatrogenic coronary dissection, perforation, or laceration. We excluded patients who have the following: (1) missing critical demographic information (i.e., age); (2) missing data for mortality; (3) age < 18; and/or (4) they had concomitant iatrogenic puncture or laceration of the coronary vessels. All coded co-morbidities were accumulated. The protocol was approved by the Institutional Review Board governing research involving human subjects at the Icahn School of Medicine at Mount Sinai. The MSHS Ethics Committee approved a waiver of documentation of informed consent; de-identified patient data was obtained from the MSHS Data Warehouse (https://msdw.mountsinai.org/).

### Baseline variables and feature selection

Feature selection to select potential variables for SCAD patients based on 2 steps. First, we conducted stepwise backward regression on all variables using the Holm-Bonferroni Method (Step-down, familywise error rates) and we chose variables for our full model when variables were *P* < 0.0004^8^. Second, we identified potential variables using clinical judgment based on previously published findings. From over 400 variables, the candidate features present on admission were selected to develop the prediction models; these demographic, clinical characteristic, comorbidity, medication, vital sign and laboratory value items are listed in Online Supplementary Table 1. The primary outcome was in-hospital mortality.

**Figure 1:**
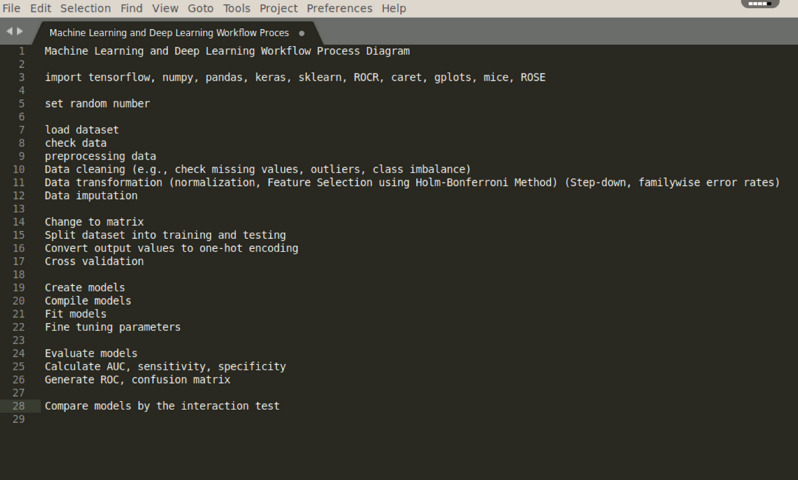
Machine learning and deep learning workflow process diagram.

**Figure 2:**
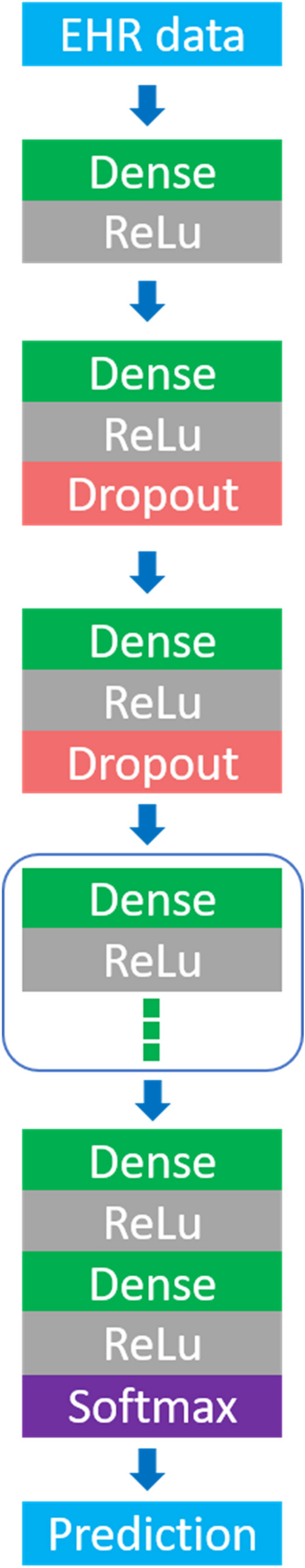
The architecture of the network.

**Table 1:** Selected baseline clinical characteristics of patients with SCAD.

Clinical characteristics	SCAD (*N* = 375)	Missing data (%)
Age (mean ± SD)	52.2 ± 12.8	0
Female	64.3%	0
White	44%	0
African-American	6.9%	0
Hispanic	4.8%	0
Asian	3.2%	0
Others/Unknown	36.3%	0
Hypertension	54.7%	0
History of smoking	5.6%	0
Overweight	2.1%	0
Secondary hypertension	0.53%	0
Malignant hypertension	1.8%	0
Chronic obstructive pulmonary disease	8.8%	0
Ischemic stroke	5.6%	0
Intracranial hemorrhage	2.1%	0
Peripheral artery disease	26.9%	0
Carotid artery disease	8.5%	0
Pulmonary hypertension	13.3%	0
Atrial fibrillation	19.7%	0
Chronic kidney disease	7.5%	0
FMD	1.9%	0
Type 1 diabetes	2.4%	0
Hyperthyroid	1.1%	0
Hypothyroid	6.4%	0
Anxiety	1.1%	0
Depression	8.5%	0
Emotional stress	5.1%	0
Migraine	1.6%	0
Rheumatoid arthritis	0.8%	0
Hypertrophic cardiomyopathy	0.5%	0
Ventricular arrhythmia	31%	0
Cardiac arrest	34.5%	0
**Vital Signs**		
Body mass index (kg/m^2^),	23.4 ± 7.9	1.50
Systolic blood pressure (mmhg)	130.6 ± 16.2	1.65
Diastolic blood pressure (mmhg)	71.2 ± 9.0	7.13
Heart rate	72.3 ± 10.2	3.60
Body temperature	78.2 ± 21.9	1.97
Respiratory rate	18.6 ± 2.8	2.13
Oxygen saturation	95.8 ± 3.8	7.68
**Lab values**		
Sodium	138.7 ± 2.6	6.56
Potassium	4.1 ± 0.4	6.61
Magnesium	2.1 ± 0.3	7.40
Chloride	103.9 ± 4.0	6.94
Blood urea nitrogen	20.5 ± 10.3	6.54
Creatinine	1.29 ± 0.6	8.62
Cholesterol	82.3 ± 31.7	6.82
HDL	43.1 ± 13.2	6.68
White blood cell count	8.5 ± 4.2	6.58
Hemoglobin	12.3 ± 1.8	6.21
C-reactive protein	15.5 ± 7.9	8.30
Platelet count	219.5 ± 62.7	6.54
Albumin	3.6 ± 0.5	7.10
**Vaccination**		
Influenza vaccination	12.8%	7.60
Medications		
NSAID use	1.1%	0
Steroid use	2.4%	0
Cannabis use disorder	1.1%	0

*NSAIDs* nonsteroidal anti-inflammatory drugs.

### Machine learning analysis

Algorithms towards diagnosis or forecasting (prognosis) of an event were based on supervised learning to predict mortality using preprocessed data and several ML and DL approaches. The ML algorithms were logistic regression, support vector machine (SVM), decision tree, random forest, K-nearest neighbors, AdaBoost and extreme gradient boosting. The DL model employed a deep neural network running Python version 3.6 (Keras) with Tensorflow backend in the high performance computing (HPC) clusters^9^. Statistical properties of continuous variables (*e.g.,* laboratory measurements) were summarized using histograms or kernel density estimation when necessary. Log-transformations were used to normalize the underlying distribution of variables. Variables with > 10% missing data were excluded and remaining missing data were addressed through imputation techniques on an individual variable basis, using R version 3.3.1 (MICE and missForest packages)^10^. Event imbalance was addressed by random over-sampling^11^. We randomly partitioned the data and repeated multiple times.

Sensitivity analyses were performed by comparing various data partitions, missing imputation vs ignoring missing data (LightGBM) and by data augmentation. In these analyses, we also examined whether results changed when limited to cases with complete unimputed data or treating event imbalance with ROSE package software. Machine-learning algorithms gain functionality from variables in the training dataset. The histogram for each clinical characteristic was normalized and analyzed separately for relationship by linear regression. Hyper-parameters, a specific learned function, was randomly tuned using several values for each parameter to derive optimum values (online Supplementary Table 2). A random forest algorithm, for example, has hyper-parameters specifying the number of branches and maximum width of each branch corresponding to the number of interactions considered in the model, whereas for the neural network, hyper-parameters control for the complexity of the model, size of the network, and how network connections are activated, or “learned”. For other prediction methods, logistic regression models did not prove viable. Hyper-parameters were tuned by cross-validated performance to minimize overfitting, searching the grid by sampling incremental combinations of multiple variables for the model, and including a parameter to control for dropping from the model variables that did not contribute to minimizing loss of functionality to identify the optimal parameters^12^. Once optimized based on the training dataset, performance of the final predictive model was assessed using the evaluation dataset. Each of several ML and DL models using tenfold cross-validation was used to estimate performance, minimize biases, and optimize hyperparameters. A summary of the workflow process for ML and DL is presented in Fig. 1. All methods were carried out in accordance with relevant guidelines and regulations. Given this particular dataset and heterogeneity in nature, we used oversampling techniques to control for dataset imbalance as well as reported balance accuracy.

**Table 2:** Comparison among machine learning and deep learning algorithms.

Model	Accuracy	Balanced accuracy	AUC (95% CI)
Deep learning	0.97	0.98	0.98 (0.97–0.99)
AdaBoost	0.94	0.61	0.95 (0.93–0.96)
Support vector machine	0.93	0.60	0.92 (0.89–0.94)
K-nearest neighbors	0.89	0.50	0.91 (0.88–0.93)
Extreme Gradient Boosting	0.95	0.54	0.90 (0.86–0.93)
Decision tree	0.79	0.53	0.78 (0.72–0.83)
Logistic regression	0.91	0.59	0.59 (0.51–0.67)
Random forest	0.93	0.52	0.50 (0.41–0.58)

### Deep learning model

The full model architecture explanation with mathematical can be found in the in online supplementary eMethod. The neural network architecture for this binary classification ($x = \left[ {x_{1} , x_{2} , \ldots , x_{n} } \right]^{T}$ adding weight and bias) by binary cross-entropy [− ylog(p) + (1 − y)log(1 − p)] consists of 15 regular fully-connected layers (using ReLU activation), two dropout layers, one after the second and third fully-connected layers, and a binary output layer (Softmax)^13, 14^. (Fig. 2). The output of the $k$ th neuron in a given layer can be written as $y_{k} = f\left( {\mathop \sum \limits_{j = 1}^{n} w_{kj} x_{j} + b_{j} } \right)$ for a layer with $n$ inputs and hence weights $w_{1}$ through $w_{n}$. Because the number of patients with SCAD was small and the mortality rate was low, a plethora of hidden layers may impair performance due to overfitting. The model was tested using an Adam optimizer with a learning rate of 0.01^15^. To optimize the model performance, the model was fine-tuned using grid search hyperparameter selection and optimally trained at 1,000 epochs^16^. Sensitivity analyses were performed using grid search for each hyperparameter selection, different data partitions, and different value of the class label. To minimize biases and optimize hyperparameters, we employed the nested cross-validation to fine-tune the model.

### Statistical analysis

Model performance was assessed from the area under the receiver-operator characteristic curve (AUC) for accuracy and adjusted for event imbalance using balancing statistics. The bootstrap technique was used to estimate confidence intervals. Performance assessments were performed using Caret, Scikit-learn and Keras software (R and Python, respectively). We compared models’ performance based on AUC values using the interaction test^17^.

## Results

Of 30,425 patients with acute coronary syndromes identified in the EHR survey, 375 (1.2%) had a diagnosis of SCAD. Overall, the mean age was 52.2 ± 12.8 years and 64.3% were women. Table 1 summarized selected baseline clinical characteristics for SCAD patients. Among these, 43 patients died during the index hospitalization (mortality 11.5%). Based on feature selections and regression analysis, predictors of in-hospital mortality in SCAD patients include elevated c-reactive protein, atrial fibrillation, hypertension, and steroid use. The best-performing DL models predicted in-hospital mortality with AUC 0.98 (95% CI 0.97–0.99) with mean accuracy 97%, balanced accuracy 98%, sensitivity 98%, and specificity 96%, compared to other ML models or logistic regressions (*P* < 0.0001). Table 2 summarizes all model performances. The AdaBoost method yielded an AUC of 0.95 (95% CI 0.93–0.96), mean accuracy 94%, balanced accuracy 61%, sensitivity 25%, and specificity 97%, compared to logistic regression model (*P* < 0.0001). The AUC with the support vector machine method was 0.92 (95% CI 0.89–0.94), mean accuracy 93%, balanced accuracy 60%, sensitivity 25%, and specificity 96%, compared to logistic regression model (*P* < 0.0001). The K-nearest neighbors method generated an AUC of 0.91 (95% CI 0.88–0.93), and had a mean accuracy of 89%, balanced accuracy 50%, sensitivity 74%, and specificity 97%, compared to logistic regression model (*P* < 0.0001). Extreme gradient boosting resulted in an AUC of 0.90 (95% CI 0.86–0.93), mean accuracy 95%, balanced accuracy 54%, sensitivity 83%, and specificity 99%, compared to logistic regression model (*P* < 0.0001). The decision tree model had an AUC of 0.78 (95% CI 0.72–0.83), mean accuracy of 79%, balanced accuracy 53%, sensitivity 87%, and specificity 35%, compared to logistic regression model (*P* < 0.0001). The conventional logistic regression model was associated with an AUC of 0.59 (95% CI 0.51–0.67), mean accuracy 91%, balanced accuracy 59%, sensitivity 25%, and specificity 94%, compared to logistic regression model (*P* < 0.0001). The random forest ML model had an AUC of 0.50 (95% CI 0.41–0.58), mean accuracy 93%, balanced accuracy 52%, sensitivity 25%, and specificity 96%. The random forest ML model had no statistical difference from logistic regression model. Table 3 summarizes all statistical comparison among DL and ML models.

**Table 3:** Comparison among machine learning and deep learning algorithms.

Model	AUC (95% CI)	Comparison	AUC (95% CI)	*P*-value
Deep learning	0.98 (0.97–0.99)	Support vector machine	0.92 (0.89–0.94)	< 0.0001
		K-nearest neighbors	0.91 (0.88–0.93)	< 0.0001
		Decision tree	0.78 (0.72–0.83)	< 0.0001
		Random forest	0.50 (0.41–0.58)	< 0.0001
		AdaBoost	0.95 (0.93–0.96)	0.001
		Extreme Gradient Boosting	0.90 (0.86–0.93)	< 0.0001
		Logistic regression	0.59 (0.51–0.67)	< 0.0001
AdaBoost	0.95 (0.93–0.96)	Extreme Gradient Boosting	0.90 (0.86–0.93)	0.01
		Logistic regression	0.59 (0.51–0.67)	< 0.0001
		Deep learning	0.98 (0.97–0.99)	0.0011
Support vector machine	0.92 (0.89–0.94)	K-nearest neighbors	0.91 (0.88–0.93)	0.58
		Decision tree	0.78 (0.72–0.83)	< 0.0001
		Random forest	0.50 (0.41–0.58)	< 0.0001
		AdaBoost	0.95 (0.93–0.96)	0.04
		Extreme Gradient Boosting	0.90 (0.86–0.93)	0.36
		Logistic regression	0.59 (0.51–0.67)	< 0.0001
		Deep learning	0.98 (0.97–0.99)	< 0.0001
K-nearest neighbors	0.91 (0.88–0.93)	Decision tree	0.78 (0.72–0.83)	< 0.0001
		Random forest	0.50 (0.41–0.58)	< 0.0001
		AdaBoost	0.95 (0.93–0.96)	0.01
		Extreme Gradient Boosting	0.90 (0.86–0.93)	0.65
		Logistic regression	0.59 (0.51–0.67)	< 0.0001
		Deep learning	0.98 (0.97–0.99)	< 0.0001
Extreme gradient boosting	0.90 (0.86–0.93)	Logistic regression	0.59 (0.51–0.67)	< 0.0001
		Deep learning	0.98 (0.97–0.99)	< 0.0001
Decision tree	0.78 (0.72–0.83)	Random forest	0.50 (0.41–0.58)	< 0.0001
		AdaBoost	0.95 (0.93–0.96)	< 0.0001
		Extreme Gradient Boosting	0.90 (0.86–0.93)	< 0.0001
		Logistic regression	0.59 (0.51–0.67)	< 0.0001
		Deep learning	0.98 (0.97–0.99)	< 0.0001
Logistic regression	0.59 (0.51–0.67)	Deep learning	0.98 (0.97–0.99)	< 0.0001
				< 0.0001
Random forest	0.50 (0.41–0.58)	AdaBoost	0.95 (0.93–0.96)	< 0.0001
		Extreme Gradient Boosting	0.90 (0.86–0.93)	< 0.0001
		Logistic regression	0.59 (0.51–0.67)	0.13
		Deep learning	0.98 (0.97–0.99)	< 0.0001

## Discussion

There are three main conclusions from the present study. First, elevated c-reactive protein, atrial fibrillation, hypertension and steroid use are important predictors of SCAD mortality. SCAD is a unique and heterogenous condition. Numerous studies suggested that SCAD is usually not associated with atherosclerosis or traditional cardiovascular risk factors (e.g., dyslipidemia, type 2 diabetes), but may be associated with connective tissue disease, autoimmune disease, stimulants, intense emotional stress, or intense physical exertion.

All the ML approaches, except the random forest model, outperformed conventional logistic regression models in predicting mortality during the index hospitalization for patients with ACS due to SCAD. Furthermore, the custom-built DL models outperformed both logistic regression and ML methods for predicting in-hospital mortality in patients with SCAD. Compared to either ML or DL, conventional logistic regression performed poorly at identifying predictors of early mortality in the population we examined. Even the relatively rudimentary decision tree ML approach, which had limited predictive power, had better predictive capacity. These observations are consistent with previous studies establishing that ML algorithms typically outperform regression models^18–20^. Deep neural network models also generally perform better than regression, reflecting the mathematical complexity and non-linearity of medical diagnosis and prognosis that defy simple parametric methodologies. In well-established diseases with causative agents, regression models can be used to estimate the effect of an independent variable on a dependent outcome (e.g., ACS directly predicts ischemic cardiomyopathy) and may be better than ML or DL methods when relationships are linear. In contrast, heterogeneous conditions with obscure predictors such as those linking SCAD to mortality require nonlinear analytic methodology that pools a large number of multidimensional variables to identify predictors of an infrequent outcome. That may explain how DL and ML models outperform regression models in select circumstances.

In this investigation, DL outperformed both regression and ML models. Although several ML models were more robust than regression models, a low-bias ML model such as boosting, which is based on methodology designed to minimize bias, may be subject to overfitting when applied to small amounts of heterogeneous data (high variance). This may explain why DL performed better than boosting. The DL model perhaps used multidimensional variables (matrix multiplication) including weight and bias, capturing a greater proportion of interactions between variables than kernel and regularization penalties in SVM, improving its performance compared to SVM. The SVMs must tune relatively fewer parameters, while DL requires multiple parameter selections, entailing complexity when applied to more than 400 clinical variables. Pathophysiological between disease and mortality are non-linear, particularly in heterogeneous conditions with low frequency events^21, 22^. This may be why a deep neural network using matrix multiplication for complex variable interactions outperformed all the ML models in the present study. To date, attempts to explain the iteration of stochastic gradient descent and cosine loss have yielded no reliable mathematical explanation as to how DL unravels such complex variable interactions^23, 24^.

In general, boosting models adjust for more parameters and are more suitable for objective function than random forests. In addition, random forests may cause biases related to different number of levels or correlated features of similar relevance. We found that boosting models (e.g., XGBoost) could potentially result in better performance than random forests perhaps due to optimal hyperparameter selection (aka hyperparameter tuning) and minimal noise.

We undertook this study as a proof-of-concept. The final DL model exhibited higher discrimination, better calibration, and greater classification accuracy than either logistic regression or ML models for predicting early mortality in patients with SCAD. We experimented with several DL models, including transfer learning, and found that the best results with the ReLU activation function, which outperformed the tanh and sigmoid activation functions. This is likely due to non-saturation of gradient, the inherent non-linearity, a reduction likelihood of vanishing gradient, and sparsity effects. Dropout layers on the upstream section of the deep learning may also help^25^. Why ReLU exhibited better convergence performance than the tanh and sigmoid activation functions is less clear^25^. These observations are consistent with earlier studies in which DL models outperformed ML algorithms when applied to other disease states^26–29^. This suggests that DL may be better suited to non-linear, low frequency outcomes such as SCAD mortality or recurrent SCAD, because it benefits from multiparametric adjustment and successive model-fitting. Since SCAD is an uncommon, heterogeneous, and poorly understood clinical entity, DL modeling uses repeated model-fitting to discern patterns that were not exposed by the other staged analysis methods.

This study has several limitations. First, ML and DL methodologies depend upon mathematical relationships between variables (e.g., variable selection algorithms), rather than medical knowledge and biological plausibility. Although we used clinical judgment to select variables and filter the algorithms, the DL decision process—the so-called “black box”-cannot be directly observed. While ML algorithms do not always yield information about effect size like the hazard ratio derived from Cox regression analysis, the contribution of individual variables can be determined and indicate signals in the data that are not causal associations and that should be interpreted cautiously pending validation in prospective epidemiological studies. Discrepancies between clinically and mathematically selected variables are complex, but DL methods such as quantum neural networks using systematic randomization of weights in each neuron rather than regularization/dropout/early stopping methods may be able to open the “black box” and shed light on these relationships.

The completeness, quality and consistency of data contribute to the success of ML algorithms. Missing value imputation may have biased the prediction algorithms, but there is at present no consensus on statistical standards to reflect this. Data quality is to some extent subjective and standards for assessment or regulation of quality control are lacking. Omission of potential confounders including localization of SCAD, severity of the coronary artery disease, proportion of intracoronary imaging, therapeutic management or proportion of PCI as well as life-style variables such as coffee consumption, sleep hygiene, emotional state or exercise could contribute to overfitting when applied to a SCAD cohort. Moreover, the temporal information of the predictors is a limitation. For instance, although we averaged lab values in laboratory values (e.g., troponin, BNP, NT-proBNP) for each subject, this may introduce biases for SCAD patients who did not present with an acute coronary syndrome or heart failure. Support for inclusion of several of the variables selected for the analysis we conducted, such as connective tissue disease and hormonal therapy, is inconsistent in the medical literature.

Although we employed pretrained models, hyper-parameter selection, and normalization, most of the models were prone to overfitting. Despite grid-searching, we sampled combinations of variables based on incremental significance, including hyper-parameters to control for dropping variables from the model that did not contribute to functionality. A key limitation of this proof of concept analysis is the small number of patients who developed the main outcome event of in-hospital mortality during the study period. Moreover, given a small number of SCAD patients, data splitting reduces the information available for development and possibly renders validation impotent. The model was trained for a particular period when survival after ACS was more frequent than demise. More training data, ultimately requiring a larger clinical sample, is needed to reduce the impact of these factors and others that detract from predictive power.

Given the rarity of the condition, diagnosis of SCAD patients is very challenging. Our findings require validation using additional clinical datasets from multiple health systems to assure generalizability to other populations with SCAD. Furthermore, while DL has been applied to other small clinical datasets^24, 30, 31^, its performance in cohorts with a small number of outcome events, such as the SCAD population we studied for in-hospital mortality, may not be generalizable to other disease states. Adoption of ML methodology in clinical practice requires multiple formal replication and validation steps, given the host of factors affecting variability of data (*e.g.,* laboratory collection, data cleaning) and models (*e.g.,* hyperparameter selection). In the future, blockchain-encrypted ML models could be shared among institutions or EHR systems to validate the models using the same environmental controls^32^.

Hyperparameter selection is prone to confounding, and the performance of each algorithm varies depending on specific variables of the databases and parameters employed^33^. There is currently no consensus around standards for reporting or interpreting ML or DL studies, limiting comparative analysis. In addition, confounders may arise in neural networks, and further methodological advances such as theoretical quantum neural network and systematic randomization within each neuron may prove more applicable than the traditional regularization methods currently employed^34^.

Overall, this is a proposed proof-of-concept work of ML and DL models for SCAD patients. Further studies are needed to validate the models and results in different populations.

## Conclusions

Elevated c-reactive protein, atrial fibrillation, hypertension and steroid use may be used as predictors of SCAD mortality. Although in this analysis a DL model was more predictive and discriminative than ML methods and logistic regression models to identify patients with heterogeneous clinical features such as SCAD, several limitations involving mathematical modeling, data structure and clinical integration must be addressed before these tools can be applied in clinical practice. Deep neural network models seem most promising for development to this purpose, but further methodological enhancements are needed to leverage data and develop valid predictors of early mortality in patients with SCAD. Data from prospective studies and randomized trials would greatly facilitate this effort to forecast clinical outcomes.

## Supplementary Information

Supplementary information.
