# Supplementary material for: Machine learning and deep learning to predict mortality in patients with spontaneous coronary artery dissection
Source: Sci Rep. 2021 Apr 26;11:8992. doi: 10.1038/s41598-021-88172-0 (PMC8076284; doi:10.1038/s41598-021-88172-0)
Supplement: Supplementary file 1 — Supplementary information. [file 41598_2021_88172_MOESM1_ESM.docx]

**Title:** Machine Learning and Deep learning to Predict Mortality in Patients with Spontaneous Coronary Artery Dissection

**Running Title:** Machine Learning and Deep learning in SCAD

**Author list:** Chayakrit Krittanawong, MD^1,2^, Hafeez Ul Hassan Virk, MD^3^, Anirudh Kumar, MD^4^, Mehmet Aydar, PhD^5^, Zhen Wang, PhD^6,7^, Matthew P Stewart, MEng^8,9^, Jonathan L Halperin, MD^2^

**Author Affiliations:**

^1^Section of Cardiology, Baylor College of Medicine, Houston, TX,

^2^The the Zena and Michael A. Wiener Cardiovascular Institute, Icahn School of Medicine at Mount Sinai, Mount Sinai Heart, New York, NY,

^3^Department of Cardiovascular Diseases, Case Western Reserve University, University Hospitals Cleveland Medical Center, Cleveland, OH

^4^Heart and Vascular Institute, Cleveland Clinic, Ohio,

^5^Department of Computer Science, Kent State University, Kent, OH

^6^Robert D. and Patricia E. Kern Center for the Science of Health Care Delivery, Mayo Clinic, Rochester, MN,

^7^Division of Health Care Policy and Research, Department of Health Sciences Research, Mayo Clinic, Rochester, MN,

^8^The Institute of Applied and Computational Sciences, Harvard University, Boston, MA,

^9^School of Engineering and Applied Sciences, Harvard University, Boston, MA, USA

**Word Count:** 2,933

**Conflict of Interest Disclosures:** none

**Online Supplementary**

Online Supplementary Method: Deep Learning Model

Online Supplementary Table 1

Online Supplementary Table 2

**Online Supplementary Method: Deep Learning Model**

Mortality prediction can be modeled as a binary classification task which takes as inputs a multivariate signal $x = \left[ x_{1}, x_{2}, \ldots, x_{n} \right]^{T}$adding weight and bias, and targeted outputs a binary variable, y ϵ {0,1}, corresponding to whether a particular patient lives or dies (mortality). Binary cross-entropy (CE) was used as the objective function to evaluate the loss between the actual and predicted outputs, given by CE = [-ylog(p)+(1-y)log(1-p)], where p is the predicted probability of survival. Cross-entropy loss increases as the predicted probability diverges from the actual label. A perfect model would have a cross-entropy loss of zero. The neural network architecture for this analysis consists of 15 regular fully-connected layers (using ReLU activation), two dropout layers, one after the second and third fully-connected layers, and a binary output layer (Softmax). Each layer in the fully connected neural network represents a matrix vector multiplication, the trainable weight parameters using backpropagation. Individual neurons in a network layer perform an affine transform consisting of multiplication by the neuron weight, $w$, following by addition of a bias value, $b$. The output of each neuron is then passed through its corresponding activation function $f$ to generate the output probability, which in input to the following network layer. This process is repeated for each neuron in the network layer, such that the output of the $k$th neuron in a given layer can be written as $y_{k}=f(\sum_{j=1}^{n} w_{kj}x_{j}+b_{j})$ for a layer with $n$inputs and hence weights $w_{1}$ through $w_{n}$. Because the number of patients with SCAD was small and the mortality rate was low, a plethora of hidden layers may impair performance due to overfitting. To minimize errors, we used 15 fully connected layers and 2 dropout layers for regularization, and scaled neuron weights by the inverse of dropout probability. The model was tested using an Adam optimizer with a learning rate of 0.01. To optimize the model performance, the model was fine-tuned using grid search hyperparameter selection and optimally trained at 1,000 epochs. Sensitivity analyses were performed using grid search for each hyperparameter selection, different data partitions, and different value of the class label.

**Online Supplementary Table 1:** Variables for models in predicting in-hospital mortality in SCAD patients

| Domains | Phenotypes |
| --- | --- |
| Demographics | Age (years), Gender (female; male), Ethnicity (Caucasian; Asian; South East Asian; African-American; Hispanic; other/mixed race; unknown) |
| Comorbidities | Hypertension, history of smoking, overweight, secondary hypertension, malignant hypertension, malignant hypertension involved heart and kidney, TIA, ischemic stroke, intracranial hemorrhage, peripheral artery disease, carotid artery disease, pulmonary hypertension, atrial fibrillation, hypertrophic cardiomyopathy, history of endocarditis, congenital heart diseases, heart failure, chronic kidney disease, liver disease, NASH, cirrhosis, asthma, chronic obstructive pulmonary disease, chronic bronchitis, pulmonary fibrosis, pneumonia, hyperthyroid, hypothyroid, adrenal insufficiency, ventricular arrhythmia, conduction defect, cardiogenic shock, cardiac arrest, anxiety, depression, emotional stress, fibromuscular dysplasia, type 1 diabetes, type 2 diabetes, mitral stenosis, mitral regurgitation, aortic regurgitation, aortic stenosis, ventricular septal defect, atrial septal defect, mitral valve prolapse, vasospasm, esophageal varices, migraine, parkinson, alzhiemer, dementia, ,yocardial bridge, systemic lupus erythematosus, celiac disease, hypertrophic cardiomyopathy, Marfan syndrome, Ehlers-Danlos syndrome, rheumatoid arthritis, celiac disease, granulomatosis with polyangiitis, sarcoidosis, polyarteritis nodosa, kawasaki disease, ankylosing spondylitis, multiple sclerosis, renal artery stenosis, cushing syndrome, primary aldosteronism, hyperparathyroidism, osteoporosis, pregnancy related conditions |
| Vital signs | Body mass index (kg/m^2^), systolic blood pressure (mmhg), diastolic blood pressure (mmhg), heart rate, body temperature, oxygen saturation, respiratory rate, pain score |
| Lab values | Sodium, potassium, magnesium, calcium, phosphorus, chloride, bicarbonate, blood urea nitrogen, creatinine, estimated GFR, fasting glucose, white blood cell count, hemoglobin, red cell distribution width, platelet count, B- type natriuretic peptide, thyroid stimulating hormone, vitamin B12, vitamin D, procalcitonin, erythrocyte sedimentation rate, C-reactive protein, lipid profile (HDL, LDL, TG, Cholesterol), iron, ferritin, hemoglobin A1C, antineutrophil cytoplasmic antibodies, antinuclear antibodies |
| Medication | Aspirin, warfarin, all novel oral anticoagulants, all nitrates, loop diuretics, K sparing diuretics, thiazide diuretics, all angiotensin converting enzyme inhibitors, all angiotensin II receptor blockers, all beta blockers, all calcium channel blockers, all nonsteroidal anti-inflammatory drugs, angiotensin receptor-neprilysin inhibitors, amphetamine abuse, cocaine abuse, cannabis use disorder, sedative hypnotic abuse, steroid use, selective estrogen receptor modulators, aromatase inhibitors |
| Vaccination | Influenza vaccination, measles, mumps, and rubella (MMR) vaccination, pneumococcal vaccination, Tdap, HPV vaccination, hepatitis B vaccination |
| Procedure | Percutaneous coronary intervention, coronary artery bypass grafting, implantable cardioverter-defibrillator, cardiac pacemaker, transcatheter aortic valve implantation, transcatheter mitral valve repair, cardiac rehabilitation, ambulatory cardiac monitoring |
| Others | Leave against medical advice |

**Online Supplementary Table 2:** list of hyper-parameters in each model

| **Algorithms** | **Parameters** |
| --- | --- |
| Deep learning | Neurons [12-256]  Number of epochs [10-1000]  Activation [softmax, relu, tanh, sigmoid]  Batch size [10-100]  Learning rate [0.001-0.3]  Momentum [0-0.9]  Dropout [0-0.9]  Optimizer [RMSprop, Adam] |
| AdaBoost | The maximum number of estimators [50-100]  Learning rate [0.1-1]  Algorithm [real boosting algorithm, discrete boosting algorithm] |
| Support vector machine | parameter C [1-5]  Kernel [linear, poly, rbf, sigmoid, precomputed]  Degree of the polynomial kernel function [3-10]  Kernel coefficient [0-0.1]  Gamma [scale, auto, float]  Float [100-500]  Tolerance [1e-3]  Kernel cache size [100-500]  parameter C of class weight [dict, balanced, none]  Maximal iterations [-1] |
| K-nearest neighbors | Number of neighbors [3-5]  Weight function [uniform, distance, callable]  Algorithm [auto, ball tree, kd tree, brute]  Leaf size [20-50]  Power parameter for the metric [2-10]  Metric [metricstr, callable, minkowski]  Metric function [dict, none] |
| Extreme Gradient Boosting | Loss function [‘deviance’, ‘exponential’]  Learning rate [0.1-0.3]  The number of boosting stages [100-500]  The fraction of samples [1.0]  The function to measure the quality of a split [friedman_mse, mse, mae]  The minimum number of samples required to split [2]  The minimum number of samples required to be at a leaf node [1]  The minimum weighted fraction of the sum total of weights [0]  Maximum depth of the individual regression estimators [3]  A node will be split if this split induces a decrease of the impurity greater than or equal to this value [0]  Threshold for early stopping [float, none]  The initial predictions [estimator, zero, none]  Maximal number of features for the best split [auto, sqrt, log2, int, float, none]  The proportion of training data to set aside as validation set for early stopping [0.1]  Tolerance for the early stopping [1e-4] |
| Decision tree | The function to measure the quality of a split [gini, entropy]  The strategy used to choose the split at each node [best, random]  The maximum depth of the tree [int, none]  The minimum number of samples required to split an internal node [2]  The minimum number of samples required to be at a leaf node [1]  The minimum weighted fraction of the sum total of weights [0]  The number of features to consider when looking for the best split [int, float, auto, sqrt, log2]  A node will be split if this split induces a decrease of the impurity greater than or equal to this value [0]  Threshold for early stopping in tree growth [0] |
| Logistic regression | The penalization [l1, l2, elasticnet, none]  Tolerance for stopping criteria [1e-4]  Inverse of regularization strength [1]  Weights associated with classes in the form [dict, balanced, none]  Algorithm to use in the optimization problem [newton-cg, lbfgs, liblinear, sag, saga]  Maximum number of iterations [10-100]  The Elastic-Net mixing parameter [0 – 1] |
| Random forest | The number of trees in the forest [10-100]  The function to measure the quality of a split [gini, entropy]  The maximum depth of the tree [none]  The minimum number of samples required to split an internal node [2]  The minimum number of samples required to be at a leaf node [1]  The minimum weighted fraction of the sum total of weights [  The number of features to consider when looking for the best split [auto, sqrt, log2]  Threshold for early stopping in tree growth [none]  Weights associated with classes in the form [balanced, balanced_subsample, dict] |
